# SkiMon: A Wireless Body Area Network for Monitoring Ski Flex and Motion during Skiing Sports

**DOI:** 10.3390/s22186882

**Published:** 2022-09-12

**Authors:** Aaron S. Crandall, Steven Mamolo, Mathew Morgan

**Affiliations:** School of Engineering and Applied Science, Gonzaga University, 502 E Boone Ave, Spokane, WA 99258, USA

**Keywords:** WBAN, outdoor sports, accelerometers, skiing, ski flex, GPS, Raspberry Pi, Body Area Network

## Abstract

Monitoring and gathering data on sporting activities holds significant promise for athletes, equipment developers, and physical fitness clinicians. Wireless Body Area Networks are being used in sporting environments as a means of gathering data, providing feedback, and helping to gain understanding of athletic activities. Applying WBANs to skiing situations, which have higher vibration, velocities, and damp environments than many other sports, can open up opportunities to understand the dynamics of skiing equipment behaviors, skiing routes on mountains, and how individuals react when skiing. To support these outcomes, a prototype WBAN-style off the shelf component system called SkiMon was proposed, implemented, and tested. The SkiMon system uses inexpensive ESP8266, Raspberry Pi, and sensor devices to gather high quality motion and location tracking data on skiers in real-world skiing conditions. By using IEEE 802.11b/g/n wireless networks, SkiMon is able to sample data at a minimum of 50 Hz, which is enough to model most ski vibration behaviors. These data results are shown to reflect ground truth 3D maps and the acceleration data comports with earlier works on ski vibration testing. Overall, a WBAN-based commodity components solution shows promise as a high quality sensor platform for tracking and modeling skiing activities.

## 1. Introduction

Wireless Body Area Networks (WBANs) hold significant capabilities in regards to sports metrics applications [1,2,3,4]. The use of WBANs for measuring the people, equipment, and environment during activities is well established. Open areas of research include sensor development, network performance, application validation, and many others.

In this work, the research team used a prototype WBAN-style solution for monitoring ski performance. Skiing has a long history of innovation, research, and material science work [5] to improve the equipment available to amateurs and professionals alike. New methods to record the behaviors of skis of varying materials, in varying conditions, and with different skiing styles have significant roles in the sport.

Based on the needs of the skiing community for new sources of ski performance, vibration, and flex data, the authors asked the question: “Is a primarily ’commodity off the shelf’ WBAN-style system capable of reliably measuring ski vibration data with sufficient resolution to allow modeling of skis in real world environments?” To answer this question, the researchers developed, implemented, and tested a new system, called SkiMon.

### 1.1. Background

In the United States, ski and snowboard resorts account for an approximately 3.1 billion USD market in 2022 [6]. The ski clothing market accounts for around 1.5 billion USD and ski equipment is around 4.2 billion USD in 2020 [7]. These industries are continually investing in making ski equipment safer, more entertaining, and more accessible to people.

Vibration and vibration damping is an ongoing area of research in ski design [8,9]. These areas of work have direct impacts on ski materials, shape, and ski capabilities with each successive generation of ski designs.

The primary goal of SkiMon was to gather high fidelity inertial measurement data from skis in real world skiing environments with a relatively off the shelf suite of parts. The measurements would need to be used to measure the behavior of the skis themselves on varying surfaces and conditions. Similar, less portable designs, have been used in past works for modeling bending stiffness and vibration in laboratory settings [10]. SkiMon would need to be able to be affixed to different types of skis, used consistently during the length of a day in wet conditions, and gather enough data to model the skis as they traversed snow at full speed.

The researchers reviewed several designs, and determined that a WBAN-style solution would be the best approach for SkiMon. Based on prior ski vibration works, such as Foss and Glenne [11], Gardiner [12], and Piziali and Mote [13], having sensors capable of capturing 50 Hz harmonics should gather most expected ski behaviors on average snow conditions. The primary considerations were bandwidth for sampling at at least 50 Hz on all inertial sensors, small sensor footprints to reduce effects on the vibration of the skis while in operation, and a large enough data storage for a full day of collection. Additionally, the system needed to be cost effective and deployable on commercial skis.

The final SkiMon system leveraged the low-cost single-board computer Raspberry Pi^TM^, the WEMOS D1^TM^ Mini ESP8266-based development board, and the Pololu^TM^ AltiMu-10 v5 inertial sensor as the key components. Physical packaging was designed and 3D printed to protect the equipment during operation. The system was developed and built in-house by the research team.

The SkiMon solution showed itself to be a solid IEEE 802.11 WBAN-style approach to data collection. It performed very well in multiple skiing tests and is usable on a wide range of commercial skis. This kind of approach can provide real-world data to ski designers and for third party reviewers of skis to evaluate claims by manufacturers.

### 1.2. Related Works

The research and engineering efforts outlined in this paper draw upon earlier works in two fields. First is the field of measuring ski vibration or vibration damping. These works focus on how to measure skis in both laboratory and field testing situations. Second is the use of commodity-embedded sensors and wireless devices to build wireless body area networks. The use of WBANs is heavily stilted towards medical monitoring applications, as so many of the closest WBAN designs are from those fields.

### 1.3. Related Ski Vibration Measurement and Analysis Works

Gosselin et al. [14] performed a robust and detailed study comparing bench and field testing on ski modeling. Their work studied both linear and torsion movements, and validated the overall results of prior works on measured vibration in skis on snow. Their work used nine IMUs per ski to build detailed physical body models. They noted that there is little prior on-snow measurements of ski behaviors, primarily because of the difficulty of deploying equipment and gathering significant datasets in real world conditions. Their results showed vibration frequencies in the range of 22 to 67 Hz.

Foss and Glenne [11] summarized several significant studies in the field of ski vibration damping. The primary focus is on reducing vibration transmitted to the skier and to increase the grip of the skis at high velocities. Their summary of various approaches to measuring ski vibration showed the main vibration range to be 10–200 Hz. Most of the key bending frequencies sit between 10 and 50 Hz, with higher frequencies measured in harder snow. Their work also showed that in on-snow testing, the fronts of the skis reliably vibrated at higher amplitudes than the backs of the skis.

Piziali and Mote [13] measured the aft-body bending of a ski using a laboratory shaker and then in the field. They focused their work on testing straight running instead of turning situations. The results showed bending modes at 9.7, 13.5, 39.7, 54.0, and 76.4 Hz in a laboratory setting. Their work with field measurements while skiing showed dominant bending frequencies between 16 and 24 Hz.

Gardiner [12] used 12 strain gauges and found vibration frequencies between 53 and 55 Hz while skiing on hard snow. Foss and Glenne performed similar measurements [11] and found vibrations around 20 Hz on soft snow and higher frequencies between 70 and 120 Hz on hard snow.

Rothemann and Schretter [15] developed an active vibration damping system for alpine skis. They used piezofibers to dampen the ski motion. Their work measured ski vibrations in the ranges of 15 to 30 Hz as the primary frequencies when bench testing. The outdoor field tests showed vibrations up to 20 Hz.

Neuworth et al. [16] used an IMU and GNSS-based sensor system to gather path and vibration data similar to SkiMon. In their work, they used IMUs affixed to the ski boots. The goal was to build algorithmic classifiers capable of using boot and GNSS (GPS-like) data to identify various skiing styles by professional skiers. Their IMU system gathered data at 54 Hz to operate with enough fidelity for successful classification.

Based on these prior works, the current SkiMon system is able to capture most key vibration frequencies on soft snow. The accelerometers used by SkiMon are capable of operating up to 315 Hz [17], so further testing to reach data collection at frequencies necessary for handling hard snow conditions would be needed.

#### Related ESP8266-Based Body Area Sensor Works

The ESP8622 chipset has been used for a variety of Internet of Things and Wireless Body Area Network (WBAN) projects. The inexpensive board has built in wifi and Bluetooth radios, is physically small, and has reasonably low power consumption. With some careful work, the board is able to enter moderately low power modes, giving it longer life in the field on batteries.

The most common applications in the research field are medical monitoring [18,19] and smart home applications [20,21]. The board’s radio has limited range, so in-home and short outdoor applications are where it is most suited. The use of a IEEE 802.11n wifi radio provides sufficient data bandwidth for most applications, including limited image/video data capabilities. Most applications use some kind of base station computer, in situ server, or smart phone as the primary hub for data aggregation and storage from the ESP8266 WBAN motes. The SkiMon architecture is similar, and based around a Raspberry Pi base station carried in a backpack while skiing.

### 1.4. SkiMon Contributions

The design, implementation, and evaluation of SkiMon contribute to the fields of WBAN applications and demonstrate the possibility of successfully leveraging commodity devices for ski vibration measurement. The components chosen are relatively inexpensive, with a total cost of around 300 USD for the whole project, and readily available from vendors. These devices form a body area network deployed for the skiing sport and tested in the field under normal skiing conditions.

The SkiMon system was tested with a target of a minimum of 50 Hz sample rates for acceleration and gyroscopic data collection. These rates cover many of the prior work’s vibration models on ski performance testing. SkiMon should then be able to gather data capable of handling ski testing in many conditions. The sensors used are capable of reaching 315 Hz, and future testing should be able to capture hard snow’s higher vibration rates.

SkiMon contributes novel ideas and approaches for both WBAN networks used in sport, and as a tool for measuring and modeling ski performance in the field.

## 2. Materials and Methods

The overall design and execution of the SkiMon project involved computing hardware, software, mechanical systems, in-field data collection, and post-processing. The project execution and data collection spanned the spring of 2021.

**Figure 1:**
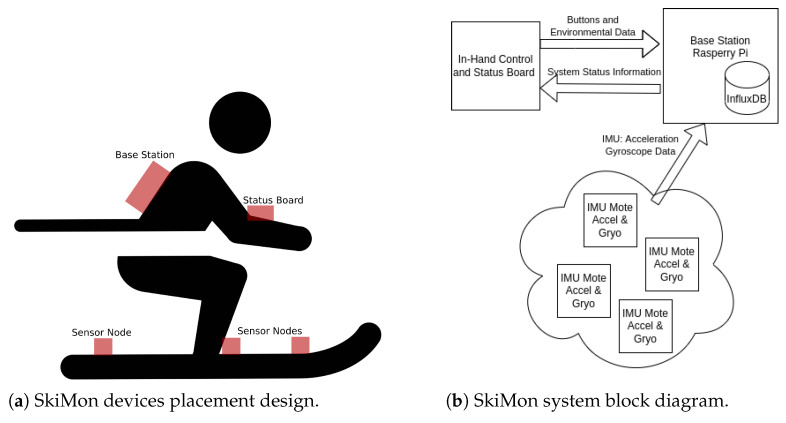
SkiMon layout and system structure.

The general design of the system is based around sensors affixed to skis worn during skiing activities. Each ski has three wireless sensor devices attached at front, middle, and back locations, as shown in Figure 1a. These devices primarily transmit accelerometer and gyroscope data to the base station carried in a backpack. The base station carried in the backpack consists of a Raspberry Pi computer, a GPS receiver, a status lights array, and a battery pack.

To allow the user to monitor the state of the equipment, a status board was designed. The status board has a network node, an OLED screen, and start/stop buttons to inject messages about when the skier begins skiing runs down the mountain. This status board was encased in a reasonably waterproof container and kept in an accessible pocket. The rough design of the devices and sensor placements are shown in Figure 1a and the overall design of the system’s block diagram is shown in Figure 1b.

### 2.1. Hardware Parts

#### 2.1.1. Base Station Raspberry Pi System

**Figure 2:**
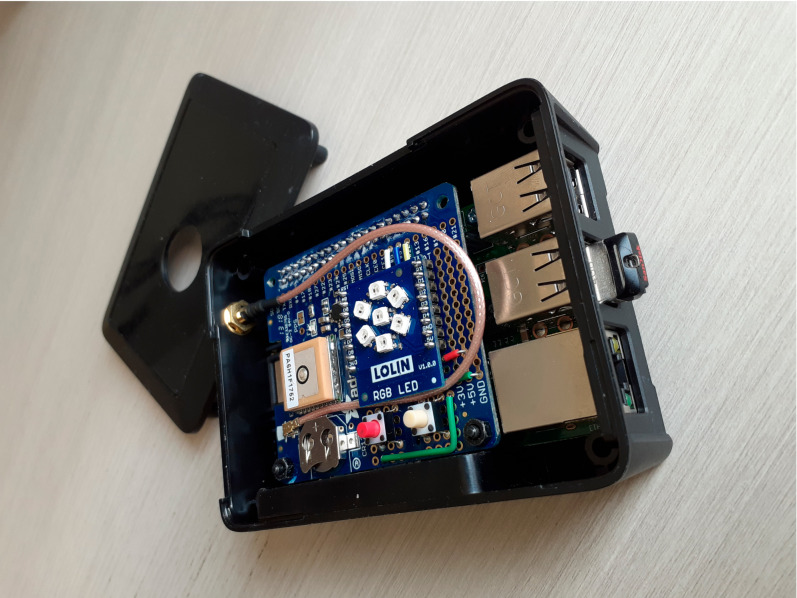
Raspberry Pi main system device showing USB flash drive, GPS hat, RGB LED status array, and GPS pigtail.

The primary data aggregation device for SkiMon is a Raspberry Pi 3 B+ board [22]. The board is running on Raspberry Pi OS, a Debian distribution port customized to the Raspberry Pi boards [23]. The data store on the RPi is a Kingston 16GB MicroSD SDC10 card and a 4 GB USB 2.0 flash drive. The RPi is housed in a simple plastic case with modifications to view the onboard RGB LEDs and an external connector for the GPS SMA antenna pigtail. The physical construction and RPi device is shown in Figure 2.

The RPi has a customized Adafruit Ultimate GPS hat [24]. This board has a LOLIN RGB LED D1 shield affixed along with a pair of momentary push switches. An external GPS antenna was affixed to the shoulder strap of the skier for the best GPS accuracy possible. Field measurements showed a GPS accuracy of around 1.5 m with this setup.

The RPi was powered by an Anker PowerCore 10,000 mAh USB battery bank. Power drain tests showed the RPi would stay consistently powered for over 18 h with this configuration.

#### 2.1.2. Inertial Motion Unit Sensor Package

The primary data sensor platform for SkiMon is the inertial measurement unit (IMU) sensor affixed to the skis. These sensors gather data using 10 degree of freedom (DOF) sensor packages. The packages chosen were Pololu AltiMU-10 v5 sensor boards. Each board has a three-axis accelerometer and gyroscope in the LSM6DS33 [17] sensor. The board provides a threex-axis LIS3MDL magnetometer [25]. Lastly, the board has a LPS25H digital barometer [26] for measuring barometric air pressure, humidity, and temperature.

The sensor package is driven by the WEMOS D1 Mini board [27]. This device uses a ESP8266 processor and wifi radio [28]. Power is provided and managed on this device using a WEMOS battery shield [29] and a 350 mAh LiPo battery.

The IMU sensor is encased in a custom designed 3D printed case using PETG filament. The IMU sensor case is made of light plastic and is gasketed to be waterproof for use in the snow. At a size of 7 × 6 × 3.2 cm, the case is small enough to fit on most standard downhill skis.

**Figure 3:**
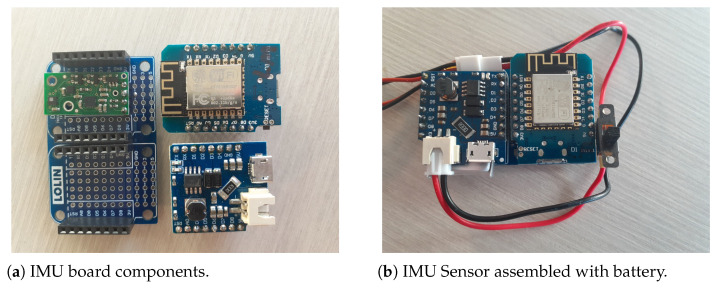

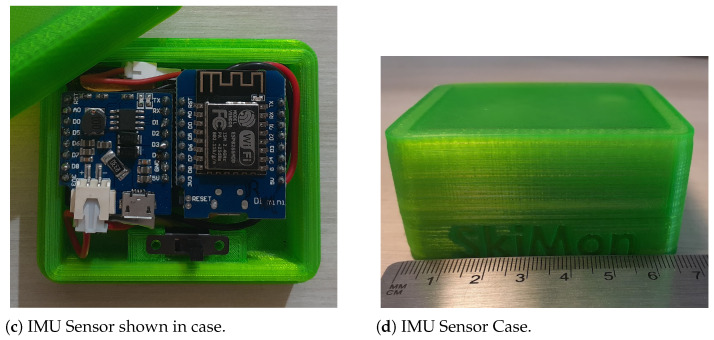
Inertial Measurement Unit WBAN ski motion sensor parts.

The case and sensor installation are shown in Figure 3c,d. Only one device fell off during testing. It was recovered and reaffixed using the 3M double sided tape and some duct tape for the rest of the testing period. The 3D CAD design for the case is available in the SkiMon Git repository [30].

#### 2.1.3. In-Hand Control and Status Board

While equipped with the SkiMon devices, a skier collecting data needs feedback about the status of the system. This includes the health of the Base Station, whether the IMU sensors are collecting data, and if the GPS has a lock for accurate location data. To provide this interface, a handheld remote control device was designed and built to provide this status information, and to inject information about the status of data collection in real time.

**Figure 4:**
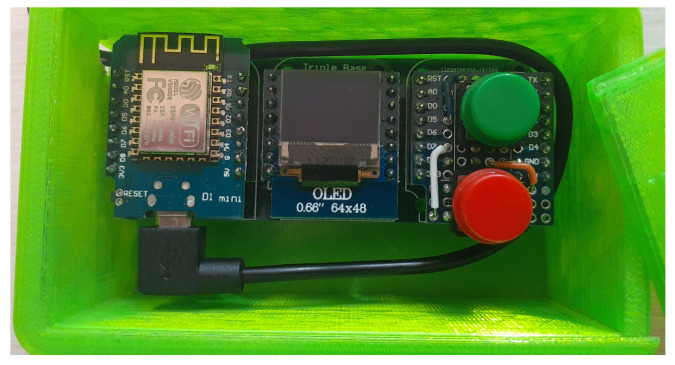
In-hand control and status board.

The Status Board device is based around a ESP8266, just as the IMU sensors are. The device is shown in Figure 4. It has a BME280 sensor for temperature and altitude information that it transmits via MQTT. Additionally, it has a small 64 × 48 OLED screen and two buttons. The whole package is mounted in a 3D printed case and powered by a USB battery back. The case is kept in a jacket pocket, and the cover is removable with ski gloves on.

The OLED screen showed the status of each IMU sensor. It also rendered the CPU load, disk space remaining, and RAM use of the Base Station.

The two buttons on board injected messages into the MQTT data collection channel. The green button sends a “Ski Run Start” message and the red button sends a “Ski Run End” message. These buttons could be pressed by the skier to make it easier to locate the start and end of ski runs in the resulting dataset.

### 2.2. Software and System Configuration Notes

The software and operating system configuration notes are available in the SkiMon GitHub repository [30]. The project has software projects for the base station, IMU motes, and status board.

#### 2.2.1. Base Station Software and Configuration

The base station main board is a Raspberry Pi running RaspiOS. All of the SkiMon system daemons were configured to run via systemd on boot. The Linux operating system has several notable project related packages installed:

- gpsd—GPS hardware daemon;
- mosquitto—MQTT server;
- influxdb—Time series database;
- hostapd—Daemon to make Linux a wireless access base station;
- Notable Python3 libraries:
  - gps3;
  - paho-mqtt;
  - rpi_ws281x, adafruit-circuitpython-neopixel, and adafruit-blinka.

##### GPS Data Collection

The GPS sensor is being monitored by gpsd at 1 Hz intervals. This interface is read by a Python3 script using the gps3 library. GPS data points are published to an onboard MQTT [31] server using the Eclipse Paho library [32].

##### Data Scribe

A system daemon to log all received data was written. This Python3 script connects to the MQTT server. All messages sent to the main SkiMon data queue are processed and stored in the influxdb database. The daemon also keeps a current state of which IMU motes are actively sending data, as well as the status of the handheld status board.

##### File Data Scribe

As an additional backup for data, a simple file logging scribe was implemented. This daemon receives all data messages via MQTT and writes them without processing to the USB flash drive attached to the base station. This scribe was kept simple so that malformed messages and system restarts would not disrupt its operation as a backup of all data in raw form.

##### Heartbeat Daemon

To ensure all MQTT clients in the SkiMon system were continually receiving data, a heartbeat daemon was implemented. This script sends one simple “heartbeat” message per second through the main data queue. All listeners using event-driven rendering or logging could rely on this message to keep them alive and connected to the network. This was especially important for the ESP8266-based motes to ensure they would not disconnect while the system was booting or being tested.

##### State Lights Daemon

**Figure 5:**
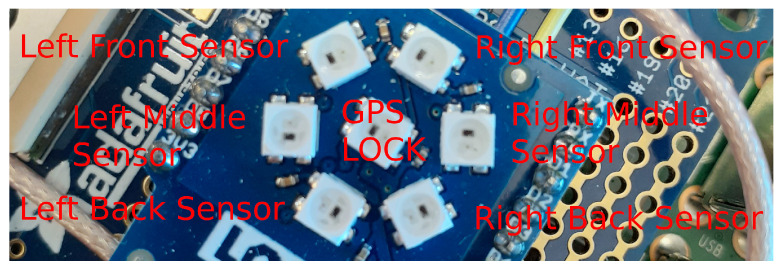
Raspberry Pi RGB LED status lights for GPS lock and motes’ connection status.

To drive the WS281x RGB LED lights on the base station, a simple status daemon was developed. This Python3 script listens to an MQTT queue for the system status. This status information included which IMU sensors were currently transmitting, whether GPS had a solid lock, and how full the disk drive was. This daemon would then render out these various status points on the seven RGB LEDs for the user to visually see what the overall system status is. The lights on the Raspberry Pi hat were provided with a Lolin RGB LED Shield for the D1 Mini. The lights and their uses are shown in Figure 5.

##### Wireless Access Base Station

The Raspberry Pi acted as a WiFi base station for the sensor network. A USB 802.11n wireless card was attached to act as the radio for the SkiMon wifi network. This system was configured using hostapd and dnsmasq. The network was available for all ESP8266 clients in the network, as well as a laptop for debugging while in the field during data collection.

The onboard wireless card was still available for connecting the Raspberry Pi to the lab research network when not in the field for testing. This allowed for quick uploads of data and to rapidly deploy new versions of the project’s code.

##### USB Flash Drive Configuration

To ensure data collection did not fill the system’s main disk partition, the USB flash disk was used as the primary data storage location. This disk was mounted at /var/lib/influxdb by permanently making an entry in /etc/fstab for it. This disk could then be filled entirely by both the scribe and file scribe daemons without impacting main operations of the computer. It also spread out the overall system I/O operations, so the full sampling rates could be achieved without having a bottleneck in the disk access.

### 2.3. Data Collection Methods

**Figure 6:**
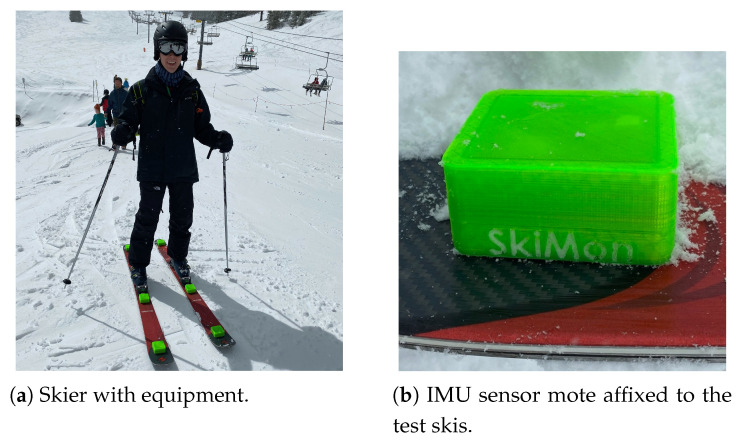
SkiMon data collection skier on Lookout Pass Mountain, day 1 of collection.

Data were collected during two days of skiing at the Lookout Pass Ski & Recreation Area on the Idaho/Montana state border. The ski runs have a summit elevation of 1722 m, with a total drop of 350 m of elevation on the longest runs. The SkiMon skier with the sensors affixed on the skis in the field are shown in Figure 6.

The skis used were a pair of 2017 Blizzard Bonafide skis, 173 cm long. These skis are considered a medium stiff flex, which were selected as likely having a more general vibration behavior while testing. The skis have a Poplar-Beech wood core with carbon fiber tips and tails to help with overall stability.

**Figure 7:**
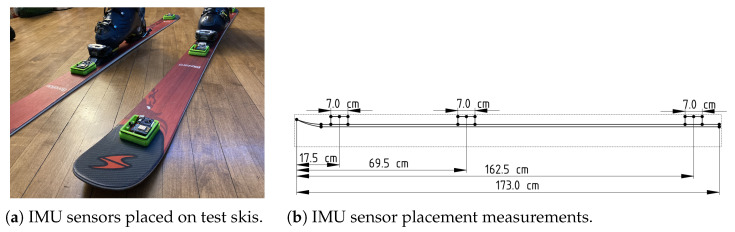
IMU sensor placement details.

The IMU sensor motes were placed on the skis with their centers at 17.5, 69.5, and 162.5 cm from the tip of the skis as shown in Figure 7b. These distances where chosen to fit with the skis’ contours. The front sensor was placed as close to the tip as could be done without overlapping the curved ski tip. The middle sensor was situated as close to the middle of the ski as could be done without interfering with the ski boot clips. The rear sensor was placed to match the setback distance from the tip being used by the front sensor. The IMU sensor itself was centered over the midline of the ski. A closeup of the sensor placement is shown in Figure 7a.

The skier wore a small backpack to hold the SkiMon base station and kept the hand control in a pocket. At the start of the day, all SkiMon components were powered on in the parking lot while putting on ski equipment and worn for the whole day.

The SkiMon equipped skier was instructed to push the “collection start” and “collection end” buttons on the hand control as they began and ended their ski runs. This injected messages into the data set with reasonable points of when collection began and ended.

The SkiMon test equipment was deployed during 16 individual descents (runs) down the mountain. The runs used a variety of routes from the summit, with both rapid and slow declines. Runs were performed on both new and compacted snow, as well as at slower and faster speeds, up to roughly 20.3 m/s.

Skiing was done in pairs with a second skier following the SkiMon equipped person for safety and to help with third party reports of anomalies such as falls or equipment loss.

On day 1 of collection, the team were able to perform seven runs. On day 2, the team performed nine runs. The same skier performed all 16 runs. All data were successfully logged on the onboard SD card and copied off after the end of collection for archiving and analysis.

## 3. Results

Data collection was performed over two days in the field. The ESP8266 and Raspberry Pi wifi networks performed reasonably well. One ESP8266 had trouble connecting consistently to the wireless network and needed to be replaced for reliability purposes.

A total of 294 min of data collection time was completed across both days. Of the total time, 53 min was during runs descending the mountain at speed. A total of 16 skiing runs were performed down the mountain, gathering a total of 5,339,206 data sample points. Most of the rest of the data collection time was either waiting for or riding the ski lifts. These numbers are summarized in Table 1.

From the 16 runs performed, they had an average descent time of 3.3 min, with a minimum of 1.1 min and a longest run of 11 min. During runs, the skier averaged 6.5 m/s with a maximum speed of 20.3 m/s.

### 3.1. Power Limitations during Collection

The base station and hand control panel were both powered with sufficiently large batteries for the duration of experimentation. The IMU motes were powered with 350 mAh LiPo batteries. No real efforts were made to reduce power consumption with sleep-based interrupts or quiesced sensor period transmission rate reduction. The result was that the batteries lasted roughly 95 min on day 1 and 80 min on day 2. This led to incomplete or entirely missing IMU data from runs #5 and #6 on day 1 and partial IMU data on runs #11 to #16 on day 2. Larger batteries and/or more energy efficient approaches should be used if longer data collection periods are desired.

### 3.2. Run Altitudes during Descent

**Figure 8:**
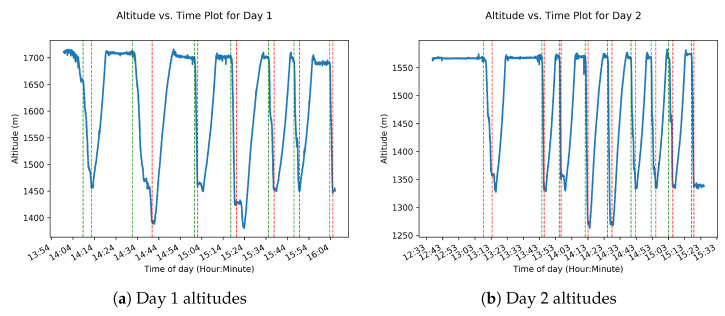
SkiMon altitude data over time. The dashed lines denote the start and end of runs, with green being starts and red being stops.

Visualization of the altitude data collected for both days shows the steepness during descents. The altitude data are rendered in Figure 8a,b. These figures show the altitude data vs. time during collection. The data come from the BME280 barometric pressure sensor, which was not properly configured with the daily sea level pressure on day 2. This led the day 2 altitudes to be calculated significantly lower than the day 1 values for the same locations.

### 3.3. GPS and Altitude Data Visualization

**Figure 9:**
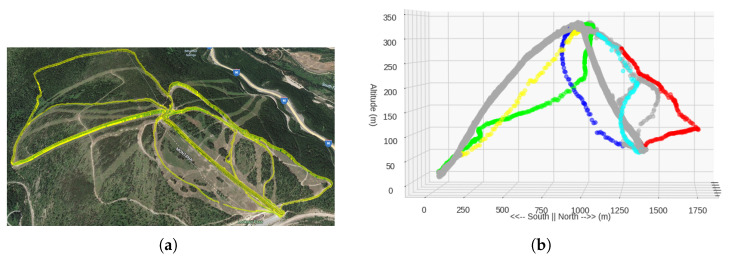
Inertial measurement unit WBAN ski motion sensor parts. (**a**) Day 1 GPS data rendered in Google Earth. (**b**) SkiMon GPS data for runs #1 through #5 on day 1. Each run is a different color, while all non-run data are in gray.

The GPS data were sampled during both days of collection. The latitude and longitude data were received at about 1 Hz. This data, when combined with the altitude data, are able to show the paths of the skier with reasonable fidelity. The first five runs, along with the non-run data, are shown in Figure 9b.

To place the GPS data in context, the same Day 1 first five runs were used to create a Google Earth KMZ file. The data for all runs are shown as rendered on Google Earth in Figure 9a. The various curves and shapes of the ski runs are visible in the data collected by the SkiMon system.

### 3.4. Ski Accelerations: Front vs. Back Sensors

To investigate the utility and quality of the collected accelerometer data as vibration data, a simple analysis was performed on the front and back accelerometer sensor outputs. The collected accelerometer data comes in the form of a three-axis value in m/s^2^.

The data for each run were discretized into 1 s windows. For each accelerometer sample in that window, the absolute magnitude of acceleration was summed up and the gravity normal subtracted. The absolute acceleration samples were then averaged for the sensor in the given time window.

**Figure 10:**
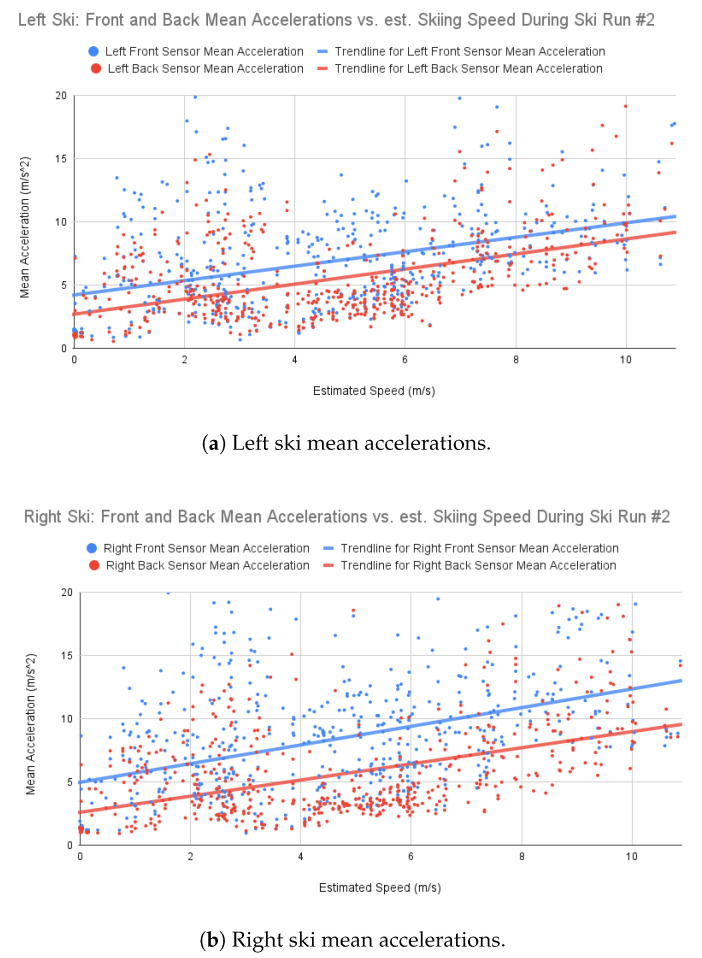
Run #2 left and right ski mean accelerations vs. speed.

These absolute mean accelerations were then plotted against the estimated velocity of the skier at that time. These results are shown in Figure 10a,b.

## 4. Discussion

### 4.1. GPS and Altitude Plotting

The GPS data provided by the SkiMon sensors could have been improved in several ways. First, the GPS sensor could have been configured for higher rates of location sampling. The device used was set to a relatively slow 1 Hz for coordinate updates. The same device can be used at up to 10 Hz, which would have provided more information about the path of the skier. Neuworth [16] used a GNSS-based system running at 1 Hz on a cellular phone for their classification, and noted that more detail would have improved their classification at higher velocities. In the SkiMon datasets during higher velocity skiing, the location plotting starts to have notable gaps once the skier exceeds 10 m/s. Increasing the rate of GPS sampling would have provided more detail during those faster skiing behaviors.

The altitudes in the SkiMon data were provided only with the barometric pressure sensor. This sensor should have been calibrated with the daily sea level air pressure to keep it more accurate between data collection sessions. This extra configuration can be done automatically with an Internet connection, but it would likely be easier to cross check the barometric pressure-based altitude against the GPS sensor output to automatically correct and calibrate it.

### 4.2. Tip vs. Tail Vibration Behaviors

Prior work done by Gosselin et al. [14] and Foss and Glenne [11] showed that the front of the skis consistently vibrated more strongly than the back ends. Most notably is Figure 5 of Foss and Glenne showing a notable difference in acceleration magnitude when comparing the front and back (tip vs. tail) of the skis. To demonstrate whether the data collected by the SkiMon system are reasonable, a similar comparison of front vs. back vibration amplitude was performed.

Because the target vibration frequencies are low, at 50 Hz, environmental noise must be considered. Issues of wind, sound, snow movements, and the skier’s movements can all influence the measured accelerations. To mitigate the influence of these sources of noise, the authors performed a relative comparison of vibration behavior instead of a more detailed one. The sensors being compared are both attached to the same ski as each other and are measuring concurrently. Noise in the environment will provide similar effects at the same samples given the relatively simple analysis provided here. More complex and isolated behavioral analysis would be needed to draw stronger conclusions about the actual modeled behavior of the skis.

The trend lines in both charts in Figure 10 show a notable difference between the amplitude of the front and back sensor measured accelerations. The front of the skis measured a stronger overall vibration amplitude during the ski run over all speeds. This result is in line with Foss and Glenne’s solution, which shows that the SkiMon WBAN-based approach could be used for similar equipment tests and evaluations.

Various kinds of snow, descent angles, velocities, turning situations, ski models and materials, and temperatures could all have impacts on the measured vibrations. The data collected for this SkiMon work are not large enough to handle the number of underlying variables in this kind of real world data collection situation, so the primary focus was on the functionality of the system for field data collection and to demonstrate similarity to prior works on expected vibrations between the front and rear of the skis. Future work on the effects of various kinds of skiing situations would need to gather a significantly larger dataset to address these variables.

## 5. Conclusions

Wireless body area networks show great promise for a wide variety of environments and needs. In this paper, the authors demonstrated the use of a ESP8266/wifi sensor network for monitoring ski routes and physical system behaviors. The goal was to use the SkiMon system to model ski behavior under varying conditions and to show how WBAN-style solutions are able to successfully work in sporting environments.

The authors were able to use off the shelf devices and simple 3D printed parts to build a successful data collection platform. The system was deployed in the field and gathered data in real-world conditions.

Aside from undersized batteries used on the IMU sensors, the system was robust and reliable, even when subjected to the vibrations and strains of skiing at speed. The data collected showed the location and trajectory of the user over time as they descended the mountain. This kind of device has a place in building models of ski slopes, evaluating ski facility design, and even being part of a safety system when tracking skiers in difficult weather conditions.

The accelerometers in the IMU sensors were able to sense and log data over the WBAN network at a rate sufficient for most kinds of ski vibration modeling. The results were in line with prior works which used more specialized and expensive tools designed for the same purpose. These new lower cost systems, such as SkiMon, should be able to increase the deployment of WBAN-style solutions for sporting measurement and modeling in the future.

## Figures and Tables

**Table 1:** Data collection days and total collection statistics.

Collection Day	Date	Runs	Length (min)	Total Data Points
Day 1	10 April 2021	7	126	2,063,454
Day 2	10 April 2021	9	168	3,275,752
Totals		16	294	5,339,206

## Data Availability

Data, source code, and other documentation for the SkiMon project is archived on GitHub.com: https://github.com/acrandal/SkiMon (accessed on 5 May 2022).

## Abbreviations

The following abbreviations are used in this manuscript:

CAD	Computer Aided Design
DOF	Degrees Of Freedom
GNSS	Global Navigation Satellite System
GPS	Global Positioning System
IMU	Inertial Measurement Unit
MQTT	Message Queuing Telemetry Transport
WBAN	Wireless Body Area Network
